# Antioxidant and Anti-Inflammatory Compounds from Edible Plants with Anti-Cancer Activity and Their Potential Use as Drugs

**DOI:** 10.3390/molecules28031488

**Published:** 2023-02-03

**Authors:** Sofía Isabel Cuevas-Cianca, Cristian Romero-Castillo, José Luis Gálvez-Romero, Zaida Nelly Juárez, Luis Ricardo Hernández

**Affiliations:** 1 Department of Chemical Biological Sciences, Universidad de las Américas Puebla, Ex Hacienda Sta. Catarina Mártir S/N, San Andrés Cholula 72810, Mexico; 2 Biotechnology Faculty, Deanship of Biological Sciences, Universidad Popular Autónoma del Estado de Puebla, 21 Sur 1103 Barrio Santiago, Puebla 72410, Mexico; 3 Chemistry Area, Deanship of Biological Sciences, Universidad Popular Autónoma del Estado de Puebla, 21 Sur 1103 Barrio Santiago, Puebla 72410, Mexico; 4 ISSTE Puebla Hospital Regional, Boulevard 14 Sur 4336, Colonia Jardines de San Manuel, Puebla 72570, Mexico

**Keywords:** cancer, natural anti-inflammatories, natural antioxidants, foods, interleukin, TNF-α

## Abstract

Food is our daily companion, performing numerous beneficial functions for our bodies. Many of them can help to alleviate or prevent ailments and diseases. In this review, an extensive bibliographic search is conducted in various databases to update information on unprocessed foods with anti-inflammatory and antioxidant properties that can aid in treating diseases such as cancer. The current state of knowledge on inflammatory processes involving some interleukins and tumor necrosis factor-alpha (TNF-α) is reviewed. As well as unprocessed foods, which may help reduce inflammation and oxidative stress, both of which are important factors in cancer development. Many studies are still needed to take full advantage of the food products we use daily.

## 1. Introduction

Human beings have used plants since ancient times to treat different diseases. Initially, these medications were administered as tinctures, teas, poultices, powders, and other herbal formulations; because of their low cost and accessibility, a large part of the world’s population uses traditional medicines for primary health care, most of which involve the use of plant extracts. On the other hand, the current drugs for clinical use in the world are or are derived from some natural product, with higher plants being the primary source of these [1]. For example, regarding anti-cancer drugs, of 247 new drugs approved in recent years, only 29 are strictly synthetic; the rest have been developed from unaltered natural products or derived from them. This is due to the benefits of natural products, which have the ability to impact multiple signaling pathways involved in the carcinogenesis process and the fewer adverse effects if we compare them with synthetic anti-cancer agents [2]. For example, methotrexate and cisplatin are associated with adverse reactions such as hair loss, gastrointestinal injury, bone marrow suppression, neurological dysfunction, and drug resistance [3,4,5].

On the other hand, aging is a process that happens day by day for any living organism; this process is characterized by the loss of physical integrity that leads to a deterioration of physiological functions and is a risk factor for the development of major human pathologies (e.g., cancer, diabetes, cardiovascular disorders, neurodegenerative diseases) that leads to inevitable death [6]. During aging, cellular processes tend to deteriorate; specifically, mitochondria produce less ATP, provoking the accumulation of free radicals and reactive oxygen species (ROS) [5,6,7,8].

In this review, inflammatory processes in cancer are discussed, focusing on cytokines IL-6, IL-10, and TNF-α, which play an important role in the inflammatory response, preventing or increasing different disorders, including cancer. Moreso, oxidative stress and its consequences are described in the proliferation of cancer in humans. Finally, edible plants that could help alleviate inflammatory processes and oxidative stress are reviewed, thus having the potential to help alleviate symptoms in cancerous processes and even as a preventive measure against cancer.

An extensive review of the literature was made using databases such as ScienceDirect^®^, Scopus^®^, PubMed^®^, Royal Society of Chemistry, MDPI, PLOS, and Google Scholar^®^. The keywords antioxidants, antioxidant activity, and antioxidant “species” with the Boolean AND were used to search for articles, and the species was specified for specific searches. For the part of inflammation in cancer, the following keywords were used: cytokines in cancer, inflammation in cancer, IL-6 in cancer, IL-10 in cancer, and TNF-α in cancer. For the part of extracts of food and edible plants with anti-inflammatory activity, the following keywords were used: edible plant extracts anti-inflammatory activity, food with anti-inflammatory activity, fruits with anti-inflammatory activity, and vegetables with anti-inflammatory activity. The search was limited to the period 2007 to November 2022; a total of 1216 articles were revised, 995 were original articles, and the rest were reviews.

## 2. Inflammation in Cancer

Cytokines are small glycoproteins that have pleiotropic effects on healthy cells, stimulating growth, differentiation, and activation. They are part of both the innate and adaptive immune systems. Depending on the microenvironment, cytokines can have pro-inflammatory, anti-inflammatory, or immunosuppressive effects. Many human cells can produce cytokines, but immune cells are primarily responsible. Cytokines’ primary function is to serve as a short-distance paracrine and autocrine communication pathway between cells and tissues [9,10].

**Figure 1:**
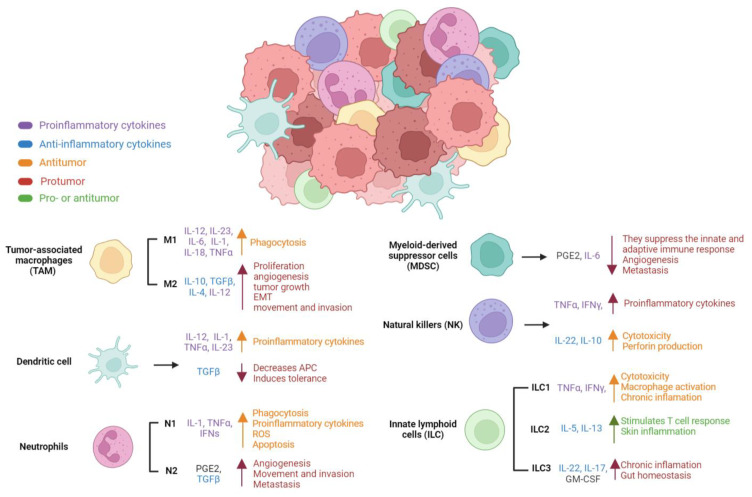
Cells of the immune system are present in the tumor microenvironment. Each cell secretes different cytokines, which can have a pro-tumorigenic, antitumorigenic, or both [11,13,20].

Pro-inflammatory cytokines are important at various stages of carcinogenesis, and inflammation has been shown to play a significant role in cancer development. Cellular differentiation, proliferation, apoptosis and growth suppression evasion, enhanced vascularity, invasion, metastasis, altered cellular metabolism, and immunological evasion are all examples of poorly regulated processes in the human body. Cancer is caused by an imbalance of pro- and anti-inflammatory mechanisms, which leads to chronic immune system activation and inflammation [11,12,13,14]. Cytokines such as tumor necrosis factor-alpha (TNF-α), interleukin-6 (IL-6), and IL-10 have been linked to cancer spread (Figure 1) [11,14,15,16,17,18,19].

### 2.1. The Tumor Microenvironment (TME)

Cytokines generated by TME cells, and some normal cells aid in invasion, tumor formation, and maintenance of cells comparable to cancer stem cells (CSC) [13]. The TME is made up of innate and adaptive immune system cells. The cytokines found in the TME interfere with immunological processes, thereby dampening the immune response and increasing tumor development [11,20]. Macrophages, dendritic cells, neutrophils, suppressor cells of myeloid origin (MDSC), natural killers (NK), and innate lymphoid cells are all members of the innate immune system (ILCs) (Figure 1) [11,13,20,21].

#### 2.1.1. Tumor-Associated Macrophages (TAM)

TAM are classified as M1 or M2 macrophages. M1s can change into M2s or vice versa depending on the microenvironment, such as inflammation, infection, hypoxia, damage, or cytokine production [22,23,24,25]. TNF-α, gamma interferon (IFN-γ), IL-12, IL-23, Toll-like receptor (TLR) ligands, and lipopolysaccharide (LPS) promote the M1 phenotype, whereas IL-14, IL-13, IL-4, IL-10, TNF-α, and TLR induce the M2 phenotype. M1 macrophages release pro-inflammatory cytokines such as IL-1, IL-6, IL-12, IL-23, IL-18, and tumor necrosis factor (TNF-α), which drive T helper cell type 1 (Th-1) responses and restrict proliferation via tissue damage induced by pro-inflammatory cytokine production. M2 macrophages generate anti-inflammatory cytokines such as IL-10, transforming growth factor beta (TGF-β), IL-4, and low amounts of IL-12, which promote immunosuppression, poor antigen presentation, tissue repair, angiogenesis, cell proliferation, Th-2 cell activity, and Th1 cell activity. M2 macrophages release chemokines that enhance cell proliferation, migration, metastasis, and epithelial-mesenchymal transition (EMT) [22,26,27].

#### 2.1.2. Dendritic Cells

Depending on the signals present in the TME, tumor-infiltrating dendritic cells (TIDC) might be immunogenic or gene tolerant. Tumors reprogram the TME to support their survival; therefore, cytokines and factors such as vascular endothelial growth factor (VEGF), IL-10, TGF-β, prostaglandin E2 (PGE-2), and TNF-α are produced in the case of TIDC that impede dendritic cells maturation and promote the gene tolerant phenotype. Depending on the TME, TIDC can operate as tumor promoters or suppressors. TIDC have been shown to serve as tumor suppressors in the early stages by secreting pro-inflammatory cytokines such as TNF-α, IL-1, IL-12, and IL-23, but as tumor promoters in the later stages [28,29,30,31,32].

#### 2.1.3. Neutrophils

Tumor-associated neutrophils (TAN) are classified as either tumor suppressors or promoters. TAN are inflammatory in the early stages of the tumor, secreting TNF-α, IL-1, and several kinds of interferons (IFNs), and immunosuppressive in the later stages, secreting TGF-β and PGE-2. N2-type TAN enhances angiogenesis, tumor growth, invasion, and metastasis, whereas N1-type TAN promotes phagocytosis, the presence of reactive oxygen species (ROS), and apoptosis [33,34,35].

#### 2.1.4. Myeloid-Derived Suppressor Cells (MDSC)

MDSC are myeloid progenitor cells, immature macrophages, immature granulocytes, and immature dendritic cells. MDSC have a role in immunosuppression in TME, including T-cell suppression and innate immune system modulation. MDSC enhances angiogenesis and metastasis by secreting IL-6 and PGE-2 [36,37,38].

#### 2.1.5. Natural Killers (NK)

To inhibit tumor cell proliferation, NK induces apoptosis via the death receptor and creates cytotoxicity via perforins and granzymes. Tumor cells in TME, on the other hand, have strategies to avoid NK by coating themselves with collagen to trick NK receptors. This leads to T-cell proliferation and expansion inhibition, strengthening their immunosuppressive features. In addition, NK has been shown to switch between an inflammatory response secreting cytokines like IFN-γ and TNF-α and an immunosuppressive response secreting cytokines like IL-22 and IL-10 depending on their environment [39,40].

#### 2.1.6. Innate Lymphoid Cells (ILC)

ILC and NK share similar features. ILC are classified into three categories: ILC1, ILC2, and ILC3. ILC1 promotes cytotoxicity, macrophage activation, and chronic inflammation by secreting the cytokines IFN-γ and TNF-α, which increase cytotoxicity, macrophage activation, and chronic inflammation. Depending on the kind of tumor, ILC2 can either promote or inhibit tumor growth. They release the cytokines IL-5 and IL-13, which increase the T-cell response and induce skin inflammation. ILC3 are carcinogenic because they secrete the immunosuppressive cytokines IL-22, IL-17, and granulocyte-macrophage colony-stimulating factor (GM-CSF) [41,42].

#### 2.1.7. Interleukin-6 (IL-6)

IL-6 is overexpressed and secreted by the tumor microenvironment (TME), which comprises various cell types such as neutrophils, macrophages, monocytes, fibroblasts, endothelial cells, lymphocytes, and tumor cells (Figure 1). Cancer-associated cells, cancer-resistant cells, and cancer stem cells are all examples of cancer cells. TNF-α is a cytokine that promotes inflammation and induces IL-6 production. Because it promotes T lymphocyte migration, expansion, activation, and differentiation, IL-6 is essential for inflammation. It also aids in differentiating B lymphocytes into plasma cells, which produce immunoglobulins. Furthermore, IL-6 is required for hematopoiesis, lipid metabolism, mitochondrial activity, and insulin resistance. IL-6 has been found to have stimulatory effects on cancer cells due to its signaling in numerous pathways that promote the cell cycle and proliferation.

When present in high concentrations, it inhibits immune system cells by suppressing IL-2 expression, decreasing T-cell activation, and encouraging lymphocyte death, preventing the immune system from detecting cancer cells [11,14,15,16,17,18,19].

#### 2.1.8. Tumor Necrosis Factor-alpha (TNF-α)

It is a pro-inflammatory cytokine expressed by macrophages and other types of cells. It is essential for the healthy operation and proliferation of NK cells, T cells, B cells, macrophages, and dendritic cells. It is also related to inflammation, immunology, and cell architecture. TNF-α, in a healthy state, is a crucial immunomodulator involved in hematopoiesis, innate immunity, dendritic cell maturation, bacterial infections, and tumor regression. In contrast, it is a major type of expressed cytokine in a variety of cancers [14,43,44,45,46].

#### 2.1.9. Interleukin-10 (IL-10)

It is an anti-inflammatory and immunosuppressive cytokine secreted by macrophages, dendritic cells, B cells, regulatory T cells, and natural killer cells (NKs) (Figure 1). Some studies indicate that IL-10 promotes tumor growth and progression. In contrast, others indicate that it aids in eradicating and suppressing angiogenesis and metastasis, both of which are required for long-term patient survival. IL-10 has three main biological activities that may contribute to the paradoxical results in a context-dependent manner: (1) promoting CD8+ T cell (CTL) proliferation and cytolytic activity, (2) inhibiting antigen presentation and production of pro-inflammatory cytokines from antigen-presenting cells (APCs), and (3) alleviating chronic inflammation via tumor-promoting effects. Regarding IL-10’s role as a tumor promoter, it is thought that it promotes immune escape from the tumor by inhibiting antigen presentation and thus decreasing the antitumor immune response in the TME. Several studies have discovered a link between IL-10 levels in the serum and tumors and a poor prognosis [15,47,48,49,50].

### 2.2. Food Extracts or Edible Plants with Anti-Inflammatory Activity

Plants are an efficient source of food and shelter, but their role as a source of medicine is underappreciated. Plants, unlike humans, are continuously and extensively exposed to natural pollutants, carcinogens, and toxic metals in nature. At the same time, plants produce various secondary metabolites, primarily used for defense and response to environmental cues such as biotic and abiotic stress [51].

Traditional medicine has used plant extracts to treat various disorders, including acute and chronic inflammation. Flavonoids are a substance in these extracts with many interesting biological properties, including anti-cancer, antimicrobial, antiviral, anti-inflammatory, immunomodulatory, and antithrombotic properties. Among these biological activities, flavonoids’ anti-inflammatory capacity has long been used in Chinese medicine via crude plant extracts. Many studies have shown that various flavonoid molecules have anti-inflammatory activity in vitro and different animal models of inflammation. Flavonoids can be found in multiple foods, including fruits, vegetables, legumes, herbs, spices, stems, flowers, tea, and red wine. They are prominent components of citrus fruits and other foods and are regularly consumed in many countries as part of a healthy diet. In Table 1, flavonoid subclasses, the names of important food flavonoids, and typical food sources are listed.

Several studies have been conducted on the anti-inflammatory activity of various foods or edible plant extracts in cancer cell lines. For example, strawberry (*Fragaria ananassa*) methanolic extracts have anti-inflammatory activity, reducing the levels of the tested inflammatory markers (NF-kB, pIkBa, TNF-α, IL-1b, IL-6, and iNOS) in RAW macrophages, which is an Abelson murine leukemia virus-induced tumor. They also discovered that the extract could promote the production of IL-10 in this study. In addition, the extract was high in vitamin C, polyphenols, and flavonoids. They also detect five anthocyanin pigments, with Pg 3-glucoside and Pg 3-malonylglucoside being the most prominent strawberry anthocyanin components [113].

The anti-inflammatory activity of lipid extract of avocado fruits and seeds (*Persea americana*) against cancer cell lines has been evaluated. Both showed activity against colon cancer cell lines (HCT116) and liver cancer cell lines (HePG2), with the seed extract showing the most activity; the LC_50_ values obtained were 22 µg/mL and 13.3 µg/mL, respectively, indicating that avocado can be considered an auspicious source of cancer drugs because it is effective against both liver and colon cancers. The lipid extract of *P. americana* fruit and seed displayed significant suppression of hepatocellular carcinoma HepG2 and colon cancer HCT116 cells vs. the reference medication sorafenib. In a chloroform/methanol extract of *P. americana* fruit and seed, oleic acid was the predominant unsaturated fatty acid. Sterol compounds were more abundant in the seed extract than in the fruit extract [114].

Mediterranean herbs such as rosemary and sage have been used for culinary and medicinal purposes for millennia. Carnosol was initially isolated from sage (*Salvia carnosa*), but we now know that rosemary also contains polyphenols like carnosol. Carnosol appears to target multiple deregulated pathways associated with inflammation and cancer, including NF-кB, apoptotic-related proteins, phosphatidylinositol-3-kinase (PI3K)/Akt, androgen, estrogen receptors, and molecular targets. Carnosol decreased LPS-stimulated nitric oxide (NO) generation in RAW cells with an IC_50_ of 9.4 µM. This compound inhibited the mitogen-activated protein kinases NF-kB, p38, and p44/42 (MAPK). Likewise, carnosol is recognized to have anticancer action against prostate, breast, skin, leukemia, and colon cancer, with an IC_50_ ranging from 5 to 82 µM [115]. Furthermore, the anti-inflammatory capacity of sage (*Salvia officinalis*) supercritical extracts was evaluated; the results showed that the extracts suppressed the production of TNF-α, IL-1, and IL-6. Camphor, borneol, and 1,8-cineole were the extract’s main components, and they all had anti-inflammatory properties. With 30 µg/mL of the extracts, the quantity of TNF-α released was significantly reduced, and TNF-α production was even lower than the basal level in non-activated cells. The supercritical extracts also show cytotoxic action against the THP-1 cell line, with LC_50_ values ranging from 66 to 80 µg/mL [116]. Similarly, studies on the anti-inflammatory activity of methanolic extracts of rosemary (*Salvia rosmarinus*) have revealed the ability to reduce NF-кB translocation and disrupt the MAPK signaling pathway. They also discovered that *Salvia rosmarinus* extracts had a potential anti-proliferative impact on breast cancer cell lines, including MCF-7 (estrogen receptor positive) and MDA-MB-231 (triple negative), with IC_50_ values ranging from 6.83 to 15.67 µg/mL against MDA-MB-231 cell line. These results are consistent with the American National Cancer Institute standards, which state that the IC_50_ level for a crude extract to be considered a prospective anticancer agent should be less than 30 µg/mL [117].

On the inflammatory mediators TNF-α and NF-кB, the anti-inflammatory activities of celery extracts, some rich in flavone aglycones and others rich in flavone glycosides, were tested. Pure flavone aglycones, like apigenin, luteolin, and chrysoeriol, and aglycone-rich extracts significantly reduced TNF-α production at concentrations between 10–50 µg/mL; they also inhibited NF-кB transcriptional activity at 25 µg/mL in RAW cells, whereas glycoside-rich extracts had no effect [118].

For a long time, the *Inonotus obliquus* mushroom has been used as a functional food and a traditional Chinese herb. Its ethanolic extract containing compounds like ergosterol, ergosterol peroxide, and trametenolic acid has shown anti-inflammatory and cytotoxic activity. Fractions from the ethanolic extract of *I. obliquus* showed an IC_50_ between 29 and 57 µg/mL against PC3, which is a prostate adenocarcinoma cancer cell line, and also the fractions showed an IC_50_ between 19 and 46 µg/mL against MDA-MB-231 which is a murine breast adenocarcinoma cell line [119].

On the other hand, studies on the ethanolic extract of ginger (*Zingiber officinale*) have revealed that it may act as an anti-cancer and anti-inflammatory agent by inhibiting NF-кB activation through suppressing pro-inflammatory cytokine, TNF-α [120].

Considering the research findings on the anti-inflammatory and anticancer activity of extracts or compounds found in food sources, we can conclude that *P. americana*, *S. carnosa*, and *S. rosmarinus* extracts are highly promising sources of cancer medications because they have the lowest IC_50_ values against various types of cancer cell lines. In addition, Carnosol, which is present in several extracts such as sage extract, is a promising molecule because it has a significant action against cell lines such as breast cancer cell lines; nevertheless, this substance requires additional research in in vivo models. Similarly, *S. rosmarinus* and *I. obliquus* extracts have anti-inflammatory and anticancer properties, making them a promising option for inhibiting the production of inflammatory cytokines in the tumor microenvironment and improving patient prognosis.

Table 2 lists various phytochemical sources or types, such as extracts, phenols, triterpenoids, saponins, lectins, polysaccharides, peptides, and other compounds, as well as the components or types of extracts and their sources.

On the other hand, Table 3 shows clinical studies undertaken by the FDA in terms of chemicals or extracts from food sources against cancer, as well as the clinical phase of the inquiry. It should be noted that only anti-inflammatory compounds or extracts with anti-cancer action were considered; there are several compounds and extracts in clinical trials for other disorders. The chemicals in this table provide a better sense of what kinds of compounds have made it from laboratory research to clinical trials, which is encouraging for other sorts of compounds derived from food.

The information shows that extracts from edible plants or foods are an excellent source of compounds with anti-inflammatory and anti-cancer activity. Therefore, it is critical to continue research into this type of extract.

### 2.3. Oxidative Stress

Excessive production and accumulation of ROS create an internal cellular imbalance known as oxidative stress, which affects different molecules found in the internal environment of cells [160]. Endogenous cellular mechanisms control cellular and extracellular redox status, such as regulating gene expression for apoptosis [161]; on the other hand, ROS influences cell signaling under homeostatic conditions [162]. Moreover, the link between oxidative stress and pathologies such as cancer has been demonstrated [158,163], as cancer cells adapt to oxidative stress by upregulating the activity of antioxidant systems such as glutathione to counteract the damaging effects of ROS [164,165].

In 2019, Cockfield and Schafer [166] concluded that some antioxidants might help tumor cells as much as they help normal cells. This is because cancer cells have redox regulation genes, suggesting that low ROS levels are required for their survival; however, the therapeutic approach may be crucial to treating this pathology. Nevertheless, in clinical trials has been shown that antioxidant consumption might be beneficial [167,168,169,170,171,172] or have no effect [167,172,173,174], as it is suggested to depend on the metabolic demand of the individual.

It is natural to think that the consumption of antioxidants as a prevention to these potential health damages is the answer to avoid or control them. Despite not being specific, it has been shown that this family of molecules can help modulate key signaling pathways for homeostasis [169,175,176]. Several molecules have antioxidant effects, such as vitamins, peptides and proteins, minerals, enzymes, and plant-derived secondary metabolites, many of which have already been characterized and evaluated [177,178,179,180].

## 3. Foods with Antioxidant Activities

Previous works have focused on identifying relevant compounds considered natural antioxidants and aids in treating specific diseases [144,181,182,183,184,185,186,187,188,189]. In this review, no mention is made of processed foods, only those in their natural state that have been characterized and have molecules with antioxidant effects in their composition.

### 3.1. Mass-Consumed Fruit with High Antioxidant Content

#### 3.1.1. Berries

These fruits are the most popular for consumption due to their antioxidant content, being fruits of the Rosaceae, Ericaceae, Grossulariaceae, and Caprifoliaceae families [190]. In addition, several studies mention properties that support the beneficial health effects of berries, such as induction and inhibition of endogenous antioxidant enzymes, impact on the cell cycle, prevention of cell lipid oxidation, free radical scavenging, and impact on cell communication [191].

In the work of Zorzi et al. [192], different berries are reported with a wide range of antioxidant capacities using TEAC, FRAP, and DPPH tests. However, results are test-dependent, and the maximum values were for blackberries and blackcurrants, considering a significant relationship between the antioxidant test and the total antioxidant compounds. This result is due to a large number of anthocyanins in the berries (greater than 50%) and ascorbic acid, although in a lower percentage [190,192].

Many bioactive compounds from different families have been found in berries, including kaempferol derivatives, quercetin, myricetin, anthocyanins such as cyanidin, delphinidin, pelargonidin, and others such as caffeic acid, coumaric acid, gallic acid, and galloyl esters. It has been shown that anthocyanins and phenolic compounds are digestible and have bioavailability in the liver and plasma. Furthermore, it has been shown that the concentration of secondary metabolites may be higher in the consumption of berries compared to the onion and other fruits, highlighting the consumption of berries of the Ericaceae family [192,193]. On the other hand, research shows that although there is availability in plasma, the amount is insufficient to estimate a positive effect on human health, as it has been shown with patients supplemented directly with quercetin who do not have significant changes. However, it is not ruled out that the protective effects observed in vitro are due to other metabolic pathways crucial in the antioxidant effect [177,190,194,195,196,197,198,199,200].

#### 3.1.2. Banana

The banana is a tropical plant and one of the world’s most popular and widely cultivated fruits; they are monocotyledonous plants belonging to the *Musa* genus of the Musaceae family [201,202]. Their nutritional and energy value are high due to their content of carbohydrates, vitamins, potassium, magnesium, and other minerals, in addition to their contribution of fiber and low amount of lipids [203]. Furthermore, a recent review includes a list of bioactive compounds in this fruit and their health benefits, with phenolic compounds acting as antioxidants [204]. On the other hand, green banana consumption has benefits related to gastrointestinal damage, glycemic/insulin metabolism, weight control, and renal and hepatic complications associated with diabetes [205].

Phenolic compounds, carotenoids, flavonoids, and biogenic amines have received attention for their particular activity in antioxidant tests. Bananas have a higher antioxidant capacity due to the number of bioactive compounds such as catechin, ferulic acid, coumaric acid, gallic acid, dopamine, and vitamin C. These molecules have been evaluated as reducing the low-density lipoprotein and other lipids oxidation. Moreso, their antioxidant capacity in assays is equivalent to lecithin and ascorbic acid in time-dependent peroxide inhibition; furthermore, this capacity increases during fruit ripening [205,206,207,208].

The compounds obtained vary according to the extraction type used. DPPH assays of different variants have resulted in IC_50_ from 0.044 to 2.15 mg/mL showing significance due to the presence of antioxidant compounds [206,209,210]. Likewise, there have been other reviews involving different banana species where antioxidant effects and in vitro biological activity are reported, which varies depending on the variant; however, the compounds are mostly similar [204,206,210,211,212].

#### 3.1.3. Apple

*Malus domestica*, belonging to the Rosaceae family, is one of the world’s most popular and important crops and fruits. Its popularity means that it is found in many dishes for consumption. Therefore, the apple has been extensively studied; important bioactive compounds, including polyphenols, polysaccharides, sterols, pentacyclic triterpenes, and organic acids, have been reported to be found in the peel and pulp of the fruit, and it has been shown that their presence is dependent on the plant part, growing season, and consumption form [213,214,215,216,217,218].

The therapeutic value of apples has been described previously, and it is related to polyphenolic content, of which effects have been found in the absorption of gastric secretions, control of intestinal biota, elimination of toxins, and diuretic effect. It can even be interpreted that several of the molecules present may have a regulatory effect on neuronal and metabolic activity [219,220,221,222,223,224,225,226]. The secondary metabolites in fruit, such as polyphenols and anthocyanins, have a high antioxidant capacity and have been described for several years; they are often better than the vitamins they contain [216]. The apple’s metabolites reflect its antioxidant activity. According to Biedrzycka and Amarowicz [227], these metabolites are mainly in the peel than in the pulp; however, it has been shown that the pulp is often a significant source of antioxidants, which are not even lost over time [216]. The production of antioxidant compounds is affected by different variables such as cultivar, variant, harvest, geographical location, storage conditions, and manner of consumption; however, the bioactive compounds are maintained or even improved when variants are compared [228,229,230,231,232].

#### 3.1.4. Citrus

*Citrus* is a genus of flowering plants called citrus, belonging to the Rutaceae family, native to tropical and subtropical areas of Southeast Asia. The orange, tangerine, and grapefruit, among others, belong to this genus. *Citrus* fruits have a peculiar fragrance due to the flavonoids and limonoids present in their peel. These fruits are good sources of vitamin C and other bioactive compounds such as flavanones, synephrine, auraptene, and limonin [233,234,235]. Although the number of bioactive compounds varies among species, there are more than 170 antioxidants reported in the *Citrus* genus highlighting phenolic compounds; the importance in different works is that phenolic acids have a higher presence in several species, whereas terpenoids have the highest antioxidant activity in different tests [236,237,238].

#### 3.1.5. Mango

*Mangifera indica*, popular as mango, belongs to the Anacardiaceae family, consisting of about 30 species of tropical fruit trees. This fruit contains a large amount of pulp and is processed to obtain various products for consumption and is recognized for its high nutritional value due to its content of vitamins, minerals, and secondary metabolites [239]. The chemical composition of mango variants around the world has been studied, and it has been reported that mango pulp is a good source of antioxidants and possesses antidiabetic, antiviral, cardiotonic, hypotensive, and anti-inflammatory properties, with mangiferin being one of the main compounds which different beneficial activities are attributed [240,241,242]. However, although mango pulp has a high content of molecules with antioxidant capacity as it has a higher amount of phytosterols and β- and 9-*cis*-β-carotene, it has been reported that the availability of these compounds varies depending on the ripeness and variant of the fruit [243,244,245,246,247,248].

#### 3.1.6. Avocado

The *Persea americana* fruit has been consumed worldwide for 50 years [249]. Some studies analyze bioactive compounds present in the pulp of this fruit and its health benefits. It has a high content of antioxidants, highlighting the content of lutein, xanthophyll, and cryptoxanthin, which represent more than 90% of total carotenes, and gallic acid, which has a capacity equivalent to Trolox in in vitro studies [250,251,252,253,254]. In addition, studies relating the consumption of this fruit in the diet and its positive effects on health, mainly in the regulation of lipoproteins and cardiovascular control, associated with the number of phytosterols and gallic acid; it has also been shown that the consumption of this fruit leads to an improvement in obtaining nutrients during digestion [255,256,257,258]. Emphasis has also been placed on the study and analysis of the waste produced by consuming this fruit and the possible window for the industrial utilization of avocado waste-based products [259,260].

#### 3.1.7. Pineapple

Pineapple (*Ananas comosus*) belongs to the Bromeliaceae family and is a tropical perennial fruit plant known worldwide, with more than 2500 species initially cultivated in South America. Pineapple possesses several bioactive compounds, such as bromelain, and is rich in vitamins A and C, flavonoids, and tannins, among other polyphenolic compounds, organic acids, and carotenoids [261]. Extracts of this fruit have been evaluated to determine their antioxidant potential with tests such as DPPH and β-carotene-linoleate, which have been reported to be effective in eliminating free radicals, especially in polar extracts such as methanolic extract. The pineapple methanolic extract has an inhibitory activity of more than 20%, according to some studies [262,263,264]; these results are related to the vitamin C and phenolic compounds content. In addition, it has also been reported that these metabolites are also related to cytotoxic activity in different cell lines [265,266].

#### 3.1.8. Watermelon

*Citrullus lanatus*, commonly known as a watermelon, belongs to the Cucurbitaceae family and is one of the world’s most cultivated vegetables in temperate zones. Lycopene, cucurbitacin, and phenolic compounds are the main bioactive compounds in watermelon with antioxidant effects [267,268]. Lycopene is the major carotenoid present in watermelon; extracts of this fruit have been shown to have the ability to scavenge free radicals in different systems and chelate metal ions, indicating that watermelon can act as a natural antioxidant through different pathways and may be a useful therapeutic agent to treat free radical-related pathological damage [269,270].

On the other hand, it was demonstrated that the antioxidant capacity of the pulp extract in the DPPH assay has a lower percentage effect (33.05%) compared to ascorbic acid (97.42%), being lower than the studies performed on peel and seeds, but higher than lycopene in tomatoes [269,271]. As in other fruits, the metabolite content varies according to environmental factors; however, in this case, the pulp has a minor amount of bioactive compounds, although these could increase depending on the type of extraction.

#### 3.1.9. Papaya

*Carica papaya* L. belongs to the family Caricaceae, which has a high distribution worldwide, making its fruit familiar. It has a high nutritional value and is rich in vitamins, minerals, and other bioactive compounds. In addition, it has antioxidant, anti-inflammatory, antimicrobial, and other activities due to carotenoids, alkaloids, flavonoids, saponins, terpenes, and tannins found in various parts of the fruit and plant. It has been reported that the seeds and pulp contain a high antioxidant potential [272,273]. The availability of these secondary metabolites has been studied, demonstrating that ripe fruit provides a higher amount of antioxidant compounds. Furthermore, the extracts have been shown to act as suppressors of pathways involved in oxidative stress and apoptosis [274,275,276]. Finally, it is worth mentioning the work of Nieto et al. suggesting using papaya residues to create dietary concentrates with antioxidant activity [277].

### 3.2. Mass-Consumed Vegetables with High Antioxidant Content

#### 3.2.1. Tomatoes

Tomatoes belong to the Solanaceae family, which includes different tomato species that are part of daily consumption, such as *Solanum lycopersicum*, *Solanum pimpinellifolium*, and *Physalis philadelphica*, among others, due to their versatility for cultivation, in addition to their high nutritional value [278,279,280]. Furthermore, tomatoes have many antioxidant compounds and are considered an important source of carotenoids, ascorbic acid, phenolic compounds, and particularly lycopene, which has been studied against cancer [281,282,283,284,285,286].

Different parts of the tomato have been evaluated, identifying many compounds; however, most of them were found in the skin and not in the pulp. Because of the content of flavonoids and phenolic compounds, the skin has antioxidant effects greater than 50% in different tests, such as DPPH and ABTS [281,287]. Moreover, different tomato species have been characterized being their components evaluated in cell lines to assess their in vitro activity, demonstrating their cytotoxic and anti-inflammatory capacity, mainly lycopene [284,288,289,290,291,292,293,294,295,296].

Biotechnological developments have resulted in different variants of tomatoes that contain more antioxidants, although their consumption has not been proven to be significantly better than regular tomatoes [297,298].

#### 3.2.2. Potatoes

The potato (*Solanum tuberosum* L.) belongs to the Solanaceae family, is a nutritious vegetable, and is rich in calories due to its high starch content. As previously reported, it also contains active phytochemicals such as β-carotene, polyphenols, and Vitamin C, among others [299]. The presence of phenols, flavonoids, and carotenoids suggests an antioxidant activity that has been evaluated in vitro, demonstrating free radical scavenging and modulation of cellular metabolism, an important mechanism being their biotransformation during digestion and intestinal metabolism, which generates metabolites and degradation products that regulate genes contributing to defense against oxidative stress [300,301,302]. However, potato by itself is not a food that provides a significant number of antioxidants. On the other hand, it is necessary to mention that, unlike other vegetables, this tuber is not usually consumed raw, so it should be considered that there may be a loss of certain compounds and an increase in others, as mentioned in the review work of [303], which takes into consideration the changes that molecules such as anthocyanins, carotenoids, and phenols may undergo.

#### 3.2.3. Carrots

*Daucus carota* L. is a vegetable belonging to the Apiaceae family; this is a world-class vegetable due to its easy cultivation in variable climates and high nutritional value. It has been reported to have diuretic, antidiarrheal, general tonic, and antianemia activity due to its bioactive compounds; among them, phenolic compounds (mainly chlorogenic acid), carotenoids (β-carotene), polyacetylenes (falcarinol), and vitamins stand out. These compounds have been studied, demonstrating their potential to improve human health due to their anti-cancer, antioxidant, anti-inflammatory, plasma lipid modification, and serotogenic effects [304]. In addition, studies have been conducted evaluating the activity of different variants of the species finding similarities in their antioxidant activity [305].

## 4. Conclusions

Consumption of anti-inflammatory or antioxidant-rich foods is far from a cure for pathophysiology involving inflammatory processes or high levels of reactive oxygen species. It has not been demonstrated that regular consumption of these foods is related to preventing diseases such as cancer; however, there is evidence that they can be beneficial when consumed as a complementary diet during some therapies. It is worth noting that the compounds synthesized by each species will vary depending on the crop, and in some cases, the compounds of interest are not found in the food’s pulp but in the seeds or shells. More research is needed to investigate signaling or metabolic pathways where natural products positively impact inflammatory and redox processes to get the most out of the diverse range of compounds that nature provides us at our fingerprints.

## Figures and Tables

**Table 1:** Subclasses, prominent food flavonoids, and common food sources.

Flavonoid Subclasses	Name of the Flavonoid	Food Source	Ref.
**Flavanols**	Catechin, gallocatechin, epicatechin	Teas, red grapes, apricots, berries, avocado, cherries, dates, figs, grapefruit, kiwifruit, mango, medlar, melon, citrus, olives, peaches, pear, pineapple, artichokes, apple, currants, persimmons, plums, beans, broccoli, and red wines	[52,53,54,55,56,57,58,59,60,61,62,63,64,65,66,67,68,69,70,71,72,73,74,75,76]
**Flavanones**	Naringenin, hesperetin, eriodictyol	Citrus foods and almonds	[56,63,77,78,79,80,81]
**Flavones**	Apigenin, luteolin	Green leafy spices, olive oil, acerola, apple, apricot, avocado, bananas, berries, cashew apple, currant, dates, figs, citrus, grapes, guava, kiwifruit, and broccoli	[56,63,82,83,84,85,86,87,88,89,90,91,92,93,94,95,96,97,98,99,100]
**Isoflavones**	Daidzein, genistein, glycitein, biochanin A	Soybeans, soy foods, and legumes	[101,102]
**Flavonols**	Kaempferol, myricetin, quercetin, isorhamnetin	Nearly ubiquitous in foods	[103,104,105,106]
**Anthocyanidins**	Cyanidin, delphinidin, pelargonidin	Red, purple and blue berries, apples, and avocados	[63,68,70,97,107,108,109,110,111,112]

**Table 2:** Anti-inflammatory effects of phytochemicals from fruits, vegetables, and food legumes.

Classes of Phytochemicals	Components or Types of Extract	Dietary Sources	Ref.
**Crude extracts**	Procyanidin extract	Grape seeds	[121]
	Fruit juice ethanol extracts	Strawberry and mulberry	[122]
	Fruit juice with pine bark extract	Pine bark	[123]
	Citrus peel extract	Citrus	[124]
	*Sambucus* and *Rubus* species seed extracts	*Sambucus* and *Rubus* species	[125]
	Ethyl acetate extract	Chinese pear	[126]
	Ethyl acetate extract	Wild bitter melons	[127]
	Aqueous extract	Mung bean	[128]
	Acetone–water extracts	Mung bean	[129,130]
	Acetone extract	Black bean	[131]
	Ethanol extract	Adzuki bean	[132]
	Crude methanolic extracts	Legumes	[133]
	Phenolic rich extracts	White kidney beans and round purple beans	[125]
	Ethanol extract	Red bean	[134]
**Phenolics**	Polyphenols	Blueberry	[135]
	Zerumbone and 3-O-methyl kaempferol	Ginger	[136]
	Punicalagin, punicalin, strictinin A, and granatin B	Pomegranate	[137,138,139]
	Narirutin	Citrus	[140]
	Flavone velutin	Acai fruit	[141]
	Anthocyanin	Black soybean	[142]
	Phenolic compounds	Navy and black bean	[143]
**Triterpenoids**	monomeric compounds	Pear	[144]
	Pentacyclic triterpenoids	Apple	[145]
**Saponins**	Soybean saponins	Soybean	[139,141,146]
	Angularin A, angulasaponins A-C, and azukisaponins III and VI	Adzuki bean	[147]
**Lectins**	Lectins	Butterfly pea	[148]
	Monocot lectin	*Canna limbata* seeds	[149]
	Lectin	*Canavalia boliviana*	[150]
	Soybean agglutinin	Soybean	[151]
**Polysaccharides**	Polysaccharide	Welsh onion	[152]
	Water-soluble polysaccharide	*Chaenomeles speciosa* fruit	[130]
**Peptides**	Bioactive peptides	Soybean	[153]
	Lunasin	Soybean	[154]
**Other compounds**	Monogalactosyldiacylglycerol	*Citrus hystrix*	[155]
	Monogalactosyldiacylglycerol	Vegetables	[156]
	Phenethyl isothiocyanate	Cruciferous vegetables	[157]
	Indole-3-carbinol	Broccoli, cabbage, cauliflower, brussels sprouts	[158]

**Table 3:** Anti-cancer effects of phytochemicals in different stages of the FDA clinical trials [159].

Type of phytochemical	Conditions	Phase
Mangosteen extract	Apoptosis in oral and cervical cancer	NA ^‡^
Grape seed proanthocyanidin extract	Breast cancer	1
Pomegranate extract	Colorectal cancer	2
Anthocyanin extract and phospholipid curcumin (cyanidin-3-glucoside from bilberry)	Colorectal adenoma	NA ^‡^
Green tea extract	Prostate cancer	2
Noni extract (*Morinda citrifolia*)	Prostate cancer	2
Ginger Root Extract	Colorectal Cancer	2
Phenethyl isothiocyanate	Lung cancer	2
Indole-3-carbinol	Breast cancer, prostate cancer *	1

* For prostate cancer studies, the status is “Recruitment Completed”; ^‡^ Not applicable (trials without FDA-defined phases).
